# A Particular Bigeminy During Atrial Tachycardia

**DOI:** 10.1007/s12471-014-0571-7

**Published:** 2014-07-02

**Authors:** C. Buttà, A. Tuttolomondo, L. Giarrusso, A. Pinto

**Affiliations:** 1 U.O.C. Medicina Interna e Cardioangiologia, Dipartimento Biomedico di Medicina Interna e Specialistica, Università degli Studi di Palermo, Palermo, Italy; 2 U.O.C. Medicina Vascolare, Dipartimento Biomedico di Medicina Interna e Specialistica, Università degli Studi di Palermo, Piazza delle Cliniche n 2, 90127 Palermo, Italy

**Fig. 1:**
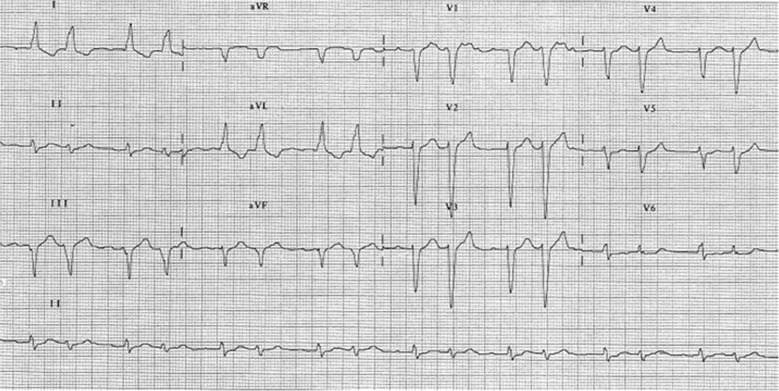
A particular bigeminy with aberrant conduction during atrial tachycardia

**Fig. 2:**
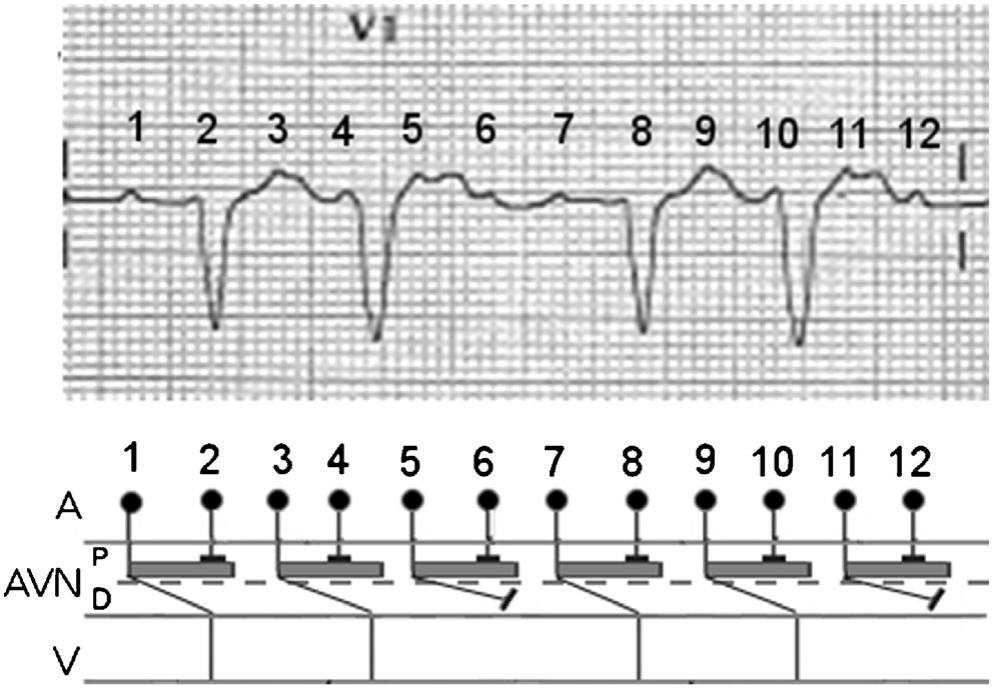
The alternating Wenckebach phenomenon visible on the V1 lead (see text for discussion). *A*, atria; *AVN*, atrioventricular node; *P*, proximal block; *D*, distal block; *V*, ventricles; 1–12 = P waves

The ECG shows clearly visible P waves only in lead V1 (Fig. 1). Regular PP intervals and an isoelectric baseline are present between the P waves, so the diagnosis is atrial tachycardia [1]. During the ECG recording, lead V1 shows 12 P waves but some of these are not visible because they are concealed by the QRS complex (Fig. 2). In lead V1, the beats following the long RR intervals are conducted by the first and the seventh P wave and the premature QRS complexes are conducted by the third and the ninth P wave because the fourth and the tenth P wave are too close to the following QRS complex to conduct the impulse. Consequently, the atrial tachycardia presents an alternating 2:1 and 4:1 conduction. However, the long RR interval is less than twice the short RR interval and the PR intervals of the premature QRS complex are longer than other PR intervals. In order to understand this mechanism, it should be considered that not just one but two constant blocks are present in the atrioventricular node: one proximal and one distal. In our case, there is a 2:1 proximal block and a 3:2 distal Wenckebach block. The distal Wenckebach block explains why the PR intervals progressively increase until a P wave is blocked causing an alternating 2:1 and 4:1 conduction; this is a rare phenomenon and it is recorded as alternating Wenckebach [2].
